# Variation in laparoscopic anti-reflux surgery across England: a 5-year review

**DOI:** 10.1007/s00464-018-6038-y

**Published:** 2018-01-24

**Authors:** Thomas R. Palser, Adam Ceney, Alex Navarro, Simon Swift, David J. Bowrey, Ian J. Beckingham

**Affiliations:** 1 Department of Upper Gastro-Intestinal Surgery, University Hospitals of Leicester NHS Trust, Leicester, UK; 2 SAPPHIRE, Department of Health Sciences, Centre for Medicine, University of Leicester, University Road, Leicester, LE1 7RH UK; 3 Methods Analytics Ltd, Sheffield Digital Campus, Electric Works, Sheffield, S1 2BJ UK; 4 Department of Hepatico-Pancreatico-Biliary surgery, Nottingham University Hospitals NHS Trust, Nottingham, UK; 5 Department of Cancer Studies, University of Leicester, Leicester, LE1 7RH UK

**Keywords:** Anti-reflux surgery, Variation

## Abstract

**Background:**

Laparoscopic anti-reflux surgery (LARS) remains central to the management of gastro-oesophageal reflux disease but the scale and variation in provision in England is unknown. The aims of this study were firstly to examine the processes and outcomes of anti-reflux surgery in England and compare them to national guidelines and secondly to explore potential variations in practice nationally and establish peer benchmarks.

**Methods:**

All adult patients who underwent LARSin England during the Financial years FY 2011/2012–FY 2016/2017 were identified in the Surgeon’s Workload Outcomes and Research Database (SWORD), which is based on the Hospital Episode Statistics (HES) data warehouse. Outcomes included activity volume, day-case rate, short-stay rate, 2- and 30-day readmission rates and 30-day re-operation rates. Funnel plots were used to identify national variation in practice.

**Results:**

In total, 12,086 patients underwent LARS in England during the study period. The operation rate decreased slightly over the study period from 5.2 to 4.6 per 100,000 people. Most outcomes were in line with national guidelines including the conversion rate (0.76%), 30-day re-operation rate (1.43%) and 2- and 30-day readmission rates (1.65 and 8.54%, respectively). The day-case rate was low but increased from 7.4 to 15.1% during the 5-year period. Significant variation was found, particularly in terms of hospital volume, and day-case, short-stay and conversion rates.

**Conclusion:**

Although overall outcomes are comparable to studies from other countries, there is significant variation in anti-reflux surgery activity and outcomes in England. We recommend that units use these data to drive local quality improvement efforts.

Gastro-oesophageal reflux disease (GORD) is a significant and increasing concern, with an estimated incidence of approximately 9–26% in European populations [1, 2]. Despite improvements in medical therapy, anti-reflux surgery remains central to its management. Evidence from randomised trials and large cohort studies indicates that surgery is safe and effective with mortality rates of < 0.3% and at least equivalent short- and long-term symptom control compared to medical management alone [3–6]. In addition, it may be more cost-effective over the longer term [6, 7].

This is set against a background of a rising interest in the development of national standards and data monitoring to drive improvements in care. In the UK, Europe and the United States, audits and quality improvement programmes have been put in place for a number of surgical conditions and procedures, such as emergency laparotomies, bariatric surgery and colorectal and oesophago-gastric cancer [8–14]. In line with this, the Association of Upper Gastro-intestinal Surgeons of Great Britain and Ireland (AUGIS) has recently both established a web-based data portal (the Surgeon’s Workload Outcomes Audit Database [15]) and published two documents which detail the service requirements and propose quality metrics for anti-reflux surgery [16, 17]. These include minimum annual surgeon volumes (at least five procedures per year with at least two surgeons per unit), a conversion to open surgery rate of < 5%, 30-day re-operation and readmission rates of < 5 and 10%, respectively, and that each unit should demonstrate a day-case rate.

However, the practice patterns and outcomes of anti-reflux surgery have never previously been examined on a national scale, either in England or elsewhere. The aims of this study therefore were firstly to examine the processes and outcomes of anti-reflux surgery in England and secondly to identify if there is variation nationally.

## Methods

Data were obtained from the NHS England Hospital Episode Statistics (HES, Copyright © 2017 Re-used with the permission of The Health and Social Care Information Centre) data warehouse using the Surgeon’s Workload Outcomes Audit Database (SWORD), a national monitoring database devised and run by Methods Analytics Ltd. together with AUGIS and the Association of Laparoscopic Surgeons of Great Britain and Ireland (ALSGBI). SWORD is a web-based portal that allows examination of HES for several different metrics in a variety of general surgical conditions (such as anti-reflux surgery, hernias, cholecystectomy, endocrine and HPB cancer surgery). Access is provided as a free member benefit to all AUGIS and ALS members. Finished Consultant Episodes are linked together such that a patient’s hospital stay encompasses all the treatment provided during that spell. Duplicates are checked and excluded.

For this study, all adult patients (those aged 18 or over) who underwent a laparoscopic anti-reflux procedure funded by the public health system (National Health Service; NHS) in an English hospital during the last five fiscal years (i.e. between 1 April 2011 and 31 March 2016) were included. Both public and private hospitals were included, although only those patients whose treatment was funded by the NHS are included in HES and hence were included in the study. Patients whose operation was performed by the open approach were excluded from the study. Eligible patients were identified by the OPCS-4.7 code G243 (anti-reflux fundoplication using abdominal approach) in association with the approach codes Y75 (Laparoscopic approach to abdominal cavity) and a primary diagnosis of K21 (Gastro-oesophageal reflux disease) or K44 (Diaphragmatic hernia). Both procedure and diagnosis codes needed to be in association with one of the laparoscopic approach codes (Y751 or Y752). Measures examined included activity volume, day-case rate, (defined as admission and discharge on the same calendar day), short-stay rate (defined as admission and discharge with only 1 or 2 overnight stays), readmission rates at 10 and 30 days and re-operation within 10 days. In order to estimate the number of procedures per 100,000 population, the Office of National Statistics mid-year population estimate for 2014 (the latest year available) was used.

The variation in the day-case rates between hospitals was assessed using funnel plots. This plot tests whether hospital rates differ significantly from the overall national rate [18]. The hospital rates are plotted on the vertical axis and the number of operations per hospital is shown on the horizontal axis. The graph also includes the mean rate for England. The two control limits indicate the ranges within which 95 and 99.8% of the rates would be expected to fall if differences from the mean English rate arose from random variation alone.

The manuscript was prepared according to the Strengthening the Reporting of Observational Studies in Epidemiology (STROBE) reporting guidelines [19].

## Results

In total, 12,086 patients underwent laparoscopic anti-reflux surgery (LARS) in England during the study period. The number of procedures was stable throughout the study period, being 2556 operations at its highest point in FY 2011/2012 and 2207 at its lowest in FY2015/2016. Based on an estimated adult English population of 49,501,761 in 2014, this corresponds to rate of anti-reflux surgery of between 4.6 and 5.2 operations per 100,000 people.

### Procedure volume

Across the study period, 57 (40%) hospitals had an average annual volume of fewer than ten procedures per year and so did not meet the AUGIS volume standard. 183 (39.5%) surgeons performing anti-reflux surgery in England had an annual volume of < 5 procedures per year. This was unchanged across the study period with the figures in FY2011/2012 and FY2015/2016 being 144 (47.2%) and 210 (61.0%), respectively. In total, 906 (7.5%) of patients were operated on by surgeons performing fewer than five procedures per year.

### Rate of conversion from laparoscopic to open

**Fig. 1:**
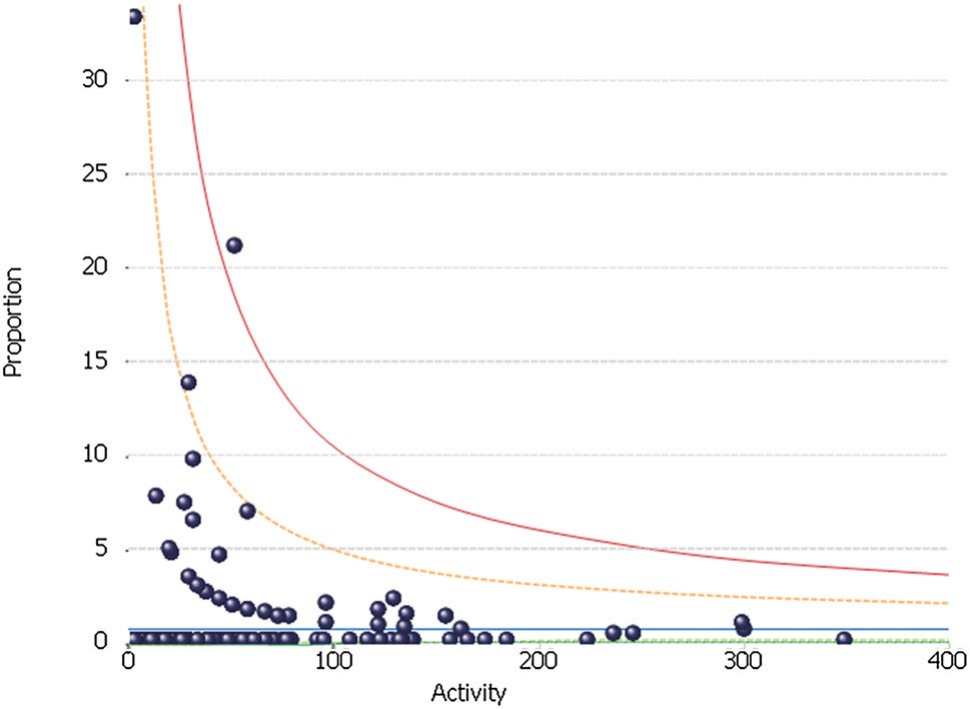
Inter-provider variation in the rate of procedures converted from laparoscopic to open: whole study period (FY2011–FY2016)

The mean conversion rate across the study period was 0.76%. This was consistent across the study period, varying from 0.89% in FY 2012/2013 to 0.45% in FY2015/2016 (Fig. 1). The conversion rate varied nationally from 0 to 33% with three units being outside the 95% control limit. Nine of the 174 hospitals had conversion rates above the 5% limit recommended by AUGIS. They were all lower volume hospitals with the largest of them having a mean annual volume of 11.6 procedures per year.

### Day-case rate

**Fig. 2:**
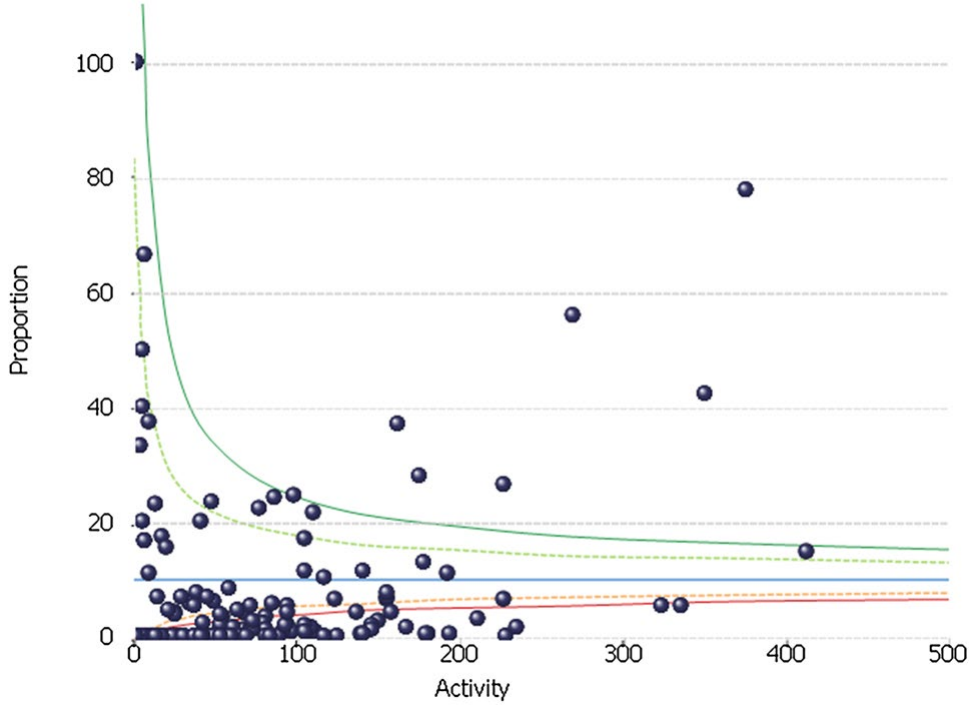
Inter-provider variation in the rate of procedures performed as a day-case: whole study period (FY2011–FY2016)

The day-case rate varied significantly between hospitals (Fig. 2). Overall, 123 (69.5%) hospitals had day-case rates below the 99.8% confidence limit (i.e. had rates significantly lower than the national mean than would be expected if the variation were due to chance alone). The overall rate fell slightly over the study period although the variation persisted, with the figure in the final year of the study being 77 (53.1)%.

In the first year of the study (FY2011/2012), 10 (7.2%) hospitals had a day-case rate of 20% or more but 109 (79.0%) hospitals did little or no day-case anti-reflux surgery (i.e. had day-case rates of < 5%). Again, the rate increased slightly across the study period although the variation remained. In the last year of the study period, 26 (18.3%) hospitals performed 20% or more of their LARS procedures as a day-case. However, 105 (73.9%) hospitals had day-case rates of < 5%. Even amongst the highest volume trusts (defined as those in the highest volume quintile), there was large variation. Three trusts performed the majority of their anti-reflux surgery as day-cases (with rates of 83.9, 74.4 and 61.8%, respectively), but fourteen high volume trusts (48.2%) had day-case rates of less than 5%. On univariate analysis, volume was not significantly associated with day-case rate (*p* = 0.064).

### Short-stay rate

**Fig. 3:**
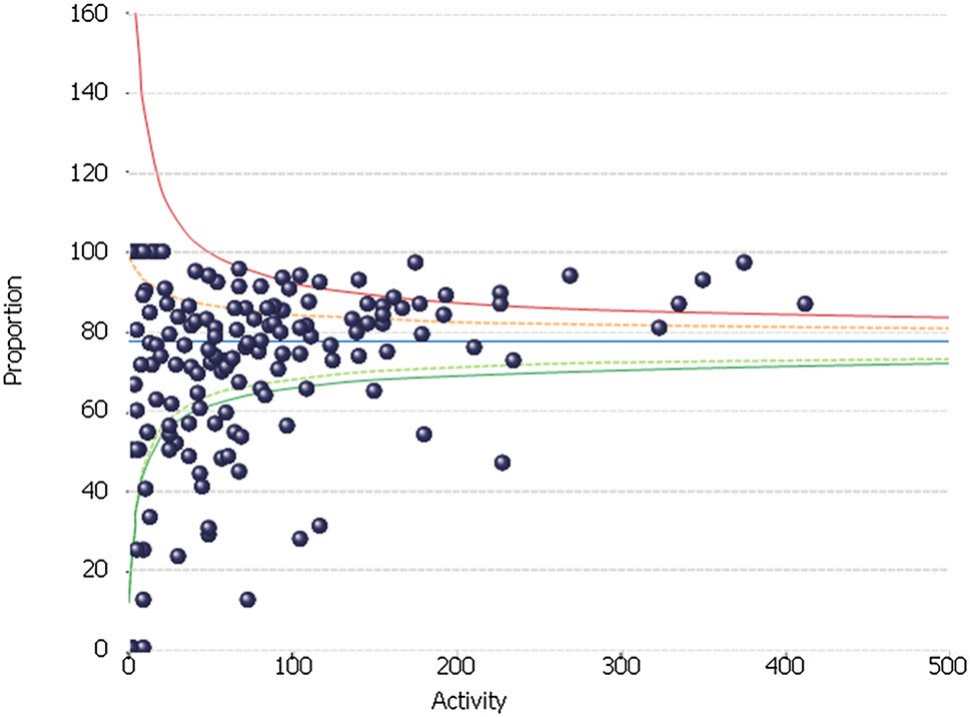
Inter-provider variation in the short-stay rate for anti-reflux surgery: whole study period (FY2011–FY2016)

Overall across the study period, 70.3% of patients were discharged within 48 h. This rate increased only slightly across the time period, from 68.8% in FY2011/2012 to 73.1% in the final year of the study. As with the day-case rate, there was significant variation in the short-stay rate which persisted across the study period (Fig. 3). In FY2011/2012, 21 (15.2%) of hospitals were below the 99.8% confidence limit. In FY 2015/2016, the figure was 14 (9.7%).

### 30-Day re-operation rates

**Fig. 4:**
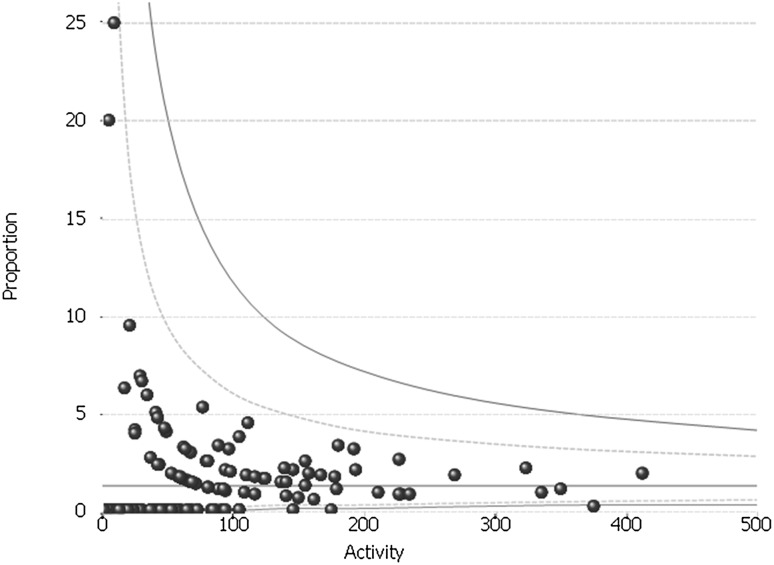
Inter-provider variation in the 30-day re-operation rate for anti-reflux surgery: whole study period (FY2011–FY2016)

The mean 30-day re-operation rate across the study period was 1.43%. This was unchanged across time (range 1.63% in FY2011/2012—1.13% in FY2015/2016 ). The rate varied between 0 and 25% although no hospitals were outside the 95% control limit. Nine hospitals had 30-day re-operation rates above the 5% AUGIS target. As with the conversion rate, these were all in lower volume hospitals (Fig. 4).

### 2- And 30-day readmission rates

**Fig. 5:**
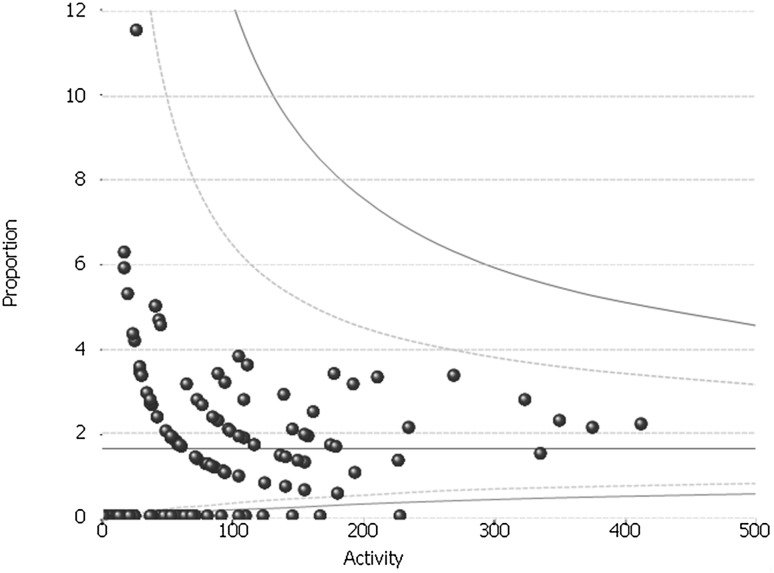
Inter-provider variation in the 2-day readmission rate for anti-reflux surgery: whole study period (FY2011–FY2016)

**Fig. 6:**
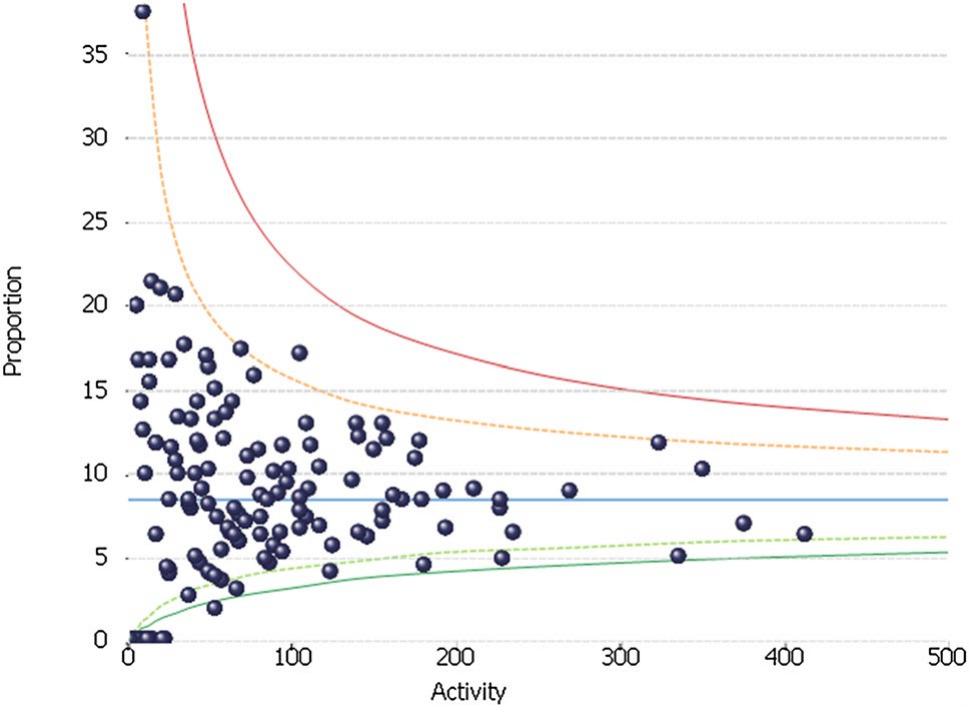
Inter-provider variation in the 30-day readmission rate for anti-reflux surgery: whole study period (FY2011–FY2016)

The mean 2- and 30-day readmission rates across the study period were 1.65 and 8.54%, respectively. This likewise was unchanged across time being 1.48 and 8.41% in FY 2011/2012 and 1.31 and 8.43% in FY2015/2016, respectively. As with the other indicators, there was significant variation nationally. The mean 2-day readmission rate across the period ranged from 0 to 11.5%, with four centres being above the 5% AUGIS target although no centres were above the 95% control limit (Fig. 5). The 30-day readmission rate varied between 0 and 37.5%. 60 Hospitals were above the 10% AUGIS target and two hospitals were above the upper 95% confidence limit (Fig. 6).

## Discussion

This study has used a national monitoring system based on administrative data to evaluate the patterns and outcomes of anti-reflux surgery in England. We found that the overall outcomes in terms of conversion to open, re-operation and readmission were comparable to other studies but that there was wide variation across the country.

The use of HES as the basis for the study (and SWORD in general) allows complete national coverage during the study period and avoids the incomplete coverage and selection bias inherent in national registries [8, 20]. It also allows examination of trends over time. HES has been used to identify treatment patterns and variation in a variety of surgical procedures including surgery for colorectal cancer [21, 22], head and neck cancer [23], breast cancer [24] and emergency surgical conditions [25]. It has been shown to be highly accurate for procedure and diagnosis codes and so the treatment patterns and outcomes here are likely to be accurate [26–28].

Amongst the potential weaknesses in the study is the fact that data quality is likely to vary between hospitals with smaller hospitals having been shown to be more affected by data quality issues [26]. We therefore cannot exclude the possibility that some of the variation observed, particularly in the smaller hospitals, was due to coding inaccuracies rather than being a real effect. However, the size of the variation observed, particularly in the day-case, short-stay and readmission rates, makes it unlikely that the findings are artefactual. This particularly applies to the national figures and trends for which even a relatively high level of inaccuracy would be unlikely to affect the overall figure significantly [29, 30].

Similarly, although HES has been shown to be highly accurate for outcomes such as readmission, re-operation and length of stay, complications such as pneumonia are reported poorly in HES. Hence, we have not included them in our study and so cannot comment on the type or occurrence rate of post-operative morbidity. Previous operations may also not be coded in HES, particularly if they occurred abroad or a long time ago before the database had matured and we cannot therefore adjust for this in the analysis. It is unlikely to contribute to the observed variation however, as the degree of previous surgery is unlikely to vary systematically.

Likewise another potential weakness is the time-lag in developing codes for novel procedures. This is potentially relevant here as new techniques such as the LINX system are slowly being introduced [31]. However, these techniques are in the early part of their introduction into UK practice and are not widely funded, so the effect of this in practice is unlikely to be large enough to affect the results. The OPCS-4 coding system is also not specific enough to differentiate between the different types of fundoplication [such as partial (Dor/Toupet) and full (Nissen’s)] so it was not possible to determine if the choice of procedure varied by hospital and if this had any effect.

More significant is the fact that national patient-reported outcome measures (PROMs) are currently only collected nationally for four procedures in the UK and this does not include anti-reflux surgery. PROMs are particularly relevant for procedures such as this in which the primary aim is symptom control and improvement of health-related quality of life. Although outcomes such as re-operation and readmission rates act as surrogates and are important outcomes in themselves, we cannot definitively comment on the “success” rates or “quality” of anti-reflux surgery in England as we lack these measures.

The overall outcomes reported here, however, are comparable to results from other studies. The overall conversion rate from laparoscopic to open of 0.76% is well below the AUGIS target of 5% and compares favourably to that published in both cohort studies and randomised controlled trials. For example, in a 20-year cohort composed of over 2200 patients in Australia, the conversion rate was 3.2% [32], whilst in the two largest RCTs it was 1.8 and 2.4% [3]. However, nine hospitals were above the AUGIS target (and one beyond the 99.8% confidence limit). Accepting that we have not adjusted for patient characteristics such as body mass index or previous abdominal surgery, there does not appear to be any obvious explanation for the higher conversion rates at these centres.

Similarly, although no trusts lay outside the statistical control limits, the variation seen in 30-day re-operation is concerning with nine hospitals lying above the 5% AUGIS benchmark. Little data exist for short-term re-operation rates after anti-reflux surgery with none of the four RCTs included in the 2010 Cochrane review explicitly reporting short-term re-operation rates [3]. The most comparable data come from a recent nationwide study from Sweden which reported outcomes on 8947 patients who underwent surgery between 1997 and 2003 [33]. In this study, the 30-day re-operation rate was lower than in our study at 0.4%. Both the overall rate and the number of trusts with much higher rates are interesting and warrant further investigation. Care must be taken, however, with over-interpretation of this outcome to avoid potentially introducing perverse incentives for surgeons to avoid re-operating when it is clinically necessary.

Interpretation of the readmission rates is more difficult. On the face of it they were higher and more variable, with 60 hospitals being above the 10% AUGIS 30-day target and two hospitals being outside the 95% control limit. Relevant data with which to compare it are sparse. Again, none of the RCTs explicitly reported it and there are no other comparable large cohort studies. A systematic review of ambulatory anti-reflux surgery described a range of 0–12.2% with an extrapolated mean of 3.5%. However, the authors comment that the evidence available for inclusion was poor quality [34]. A cohort study from the same group containing approximately 300 patients had a 30-day readmission rate of 8% [35], whilst a smaller cohort study of 113 patients who underwent day-case anti-reflux surgery in Sheffield had a rate of 3.5% [36]. The readmission rates may be confounded by centres in the early phase of introducing ambulatory anti-reflux surgery, in which readmission rates may be appropriately higher due to caution during introduction of the new protocols. Against this however, we did not find any correlation between a unit’s unadjusted 30-day readmission rate and its day-case rate.

Due partly to the faster recovery offered by the laparoscopic approach, allied to the rising emphasis on reducing healthcare costs, there is increasing interest in reducing hospital stay and increasing the number of procedures performed as a day-case. Several single-centre case series have been published which have indicated that day-case LARS can be performed safely in selected patients, with similar post-operative morbidity and mortality to those who undergo an inpatient stay, although no randomised trials have been carried out [34–38]. The one prospective study that examined cost found an estimated saving of 2367 Euros (assuming day of surgery admission as is standard UK practice) [35]. Our study has reinforced these findings by demonstrating no association between a unit’s day-case or short-stay rate and the readmission or re-operation rates.

We found that although the practice of day-case (or ambulatory) anti-reflux surgery increased nationally, with the overall rate doubling during the 5-year study period, the overall rate remained low (at 15.1% in the final year of the study) and practice variation remained widespread. A similar picture was found with the short-stay rates. Although the data are not adjusted for potential confounders such as age, comorbidity or ethnicity, the very large degree of variation observed suggests that a difference in local practice and protocols is the underlying reason. Given the potential advantages and cost savings of ambulatory or short-stay surgery, we believe that this highlights a potential area of improvement. A potential driver of this could be if the incentivised tariff system (whereby hospitals are paid more if a patient undergoes surgery as a day-case or short-stay) was extended to anti-reflux surgery.

Finally, a large proportion of operations were performed by surgeons performing fewer than the recommended number of five procedures per year. The volume outcome relationship has been established in a wide range of procedures although disputes do remain. It is interesting to note that the funnel plots in this study indicate a trend to better outcomes in the higher volume centres. As stated earlier, this could be due to poorer coding in smaller hospitals (although that in itself is relevant and it has been argued that coding is a clinical responsibility in any case).

However, it may be a real reflection of treatment and experience. A study using the National Inpatient Sample [39] divided hospitals into terciles according to their annual volume of anti-reflux surgery. They found significantly increased complication rates, length of stay and cost in the low-volume tercile compared to the higher volume tercile. The cost difference they estimated to be between $2700 and $3200.

We did not explicitly test the volume hypothesis here as the number of unmeasured confounders was too great. Nonetheless, both the overall rate and the trends observed in the funnel plots are interesting and raise questions about service organisation. Upper Gastro-Intestinal Cancer Services have undergone extensive re-organisation and centralisation in the UK and elsewhere over the last two decades, in association with which the outcomes of surgery have improved significantly. It is important to ensure that benign surgery is not neglected and is likewise performed by experienced clinical teams working in concert with adjacent smaller hospitals if necessary. The use of databases and portals such as SWORD and HES will be important in monitoring and driving this service organisation.

This study has examined the patterns and certain outcomes of anti-reflux surgery in England. The overall results are comparable to those found in studies from other Western countries but the variation observed is notable. The variability in the day-case and short-stay rates in particular indicate an area of potential improvement. We recommend that services both in the UK and internationally use these figures as a benchmark with which to compare and improve their own outcomes. These results further demonstrate the value of national administrative databases in examining variations in care and driving service improvement.
